# Development of a new 7BS.7HL winter wheat-winter barley Robertsonian translocation line conferring increased salt tolerance and (1,3;1,4)-β-D-glucan content

**DOI:** 10.1371/journal.pone.0206248

**Published:** 2018-11-05

**Authors:** Edina Türkösi, Eva Darko, Marianna Rakszegi, István Molnár, Márta Molnár-Láng, András Cseh

**Affiliations:** 1 Department of Plant Genetic Resources, Agricultural Institute, Centre for Agricultural Research, Hungarian Academy of Sciences, Martonvásár, Hungary; 2 Department of Plant Physiology, Agricultural Institute, Centre for Agricultural Research, Hungarian Academy of Sciences, Martonvásár, Hungary; 3 Cereal Breeding Department, Agricultural Institute, Centre for Agricultural Research, Hungarian Academy of Sciences, Martonvásár, Hungary; 4 Maize Breeding Department, Agricultural Institute, Centre for Agricultural Research, Hungarian Academy of Sciences, Martonvásár, Hungary; 5 Molecular Breeding Department, Agricultural Institute, Centre for Agricultural Research, Hungarian Academy of Sciences, Martonvásár, Hungary; Institute of Genetics and Developmental Biology Chinese Academy of Sciences, CHINA

## Abstract

Interspecific hybridization between bread wheat (*Triticum aestivum*, 2n = 42) and related species allows the transfer of agronomic and quality traits, whereby subsequent generations comprise an improved genetic background and can be directly applied in wheat breeding programmes. While wild relatives are frequently used as sources of agronomically favourable traits, cultivated species can also improve wheat quality and stress resistance. A salt-tolerant ‘Asakaze’/‘Manas’ 7H disomic addition line (2n = 44) with elevated β-glucan content, but with low fertility and an unstable genetic background was developed in an earlier wheat-barley prebreeding programme. The aim of the present study was to take this hybridization programme further and transfer the favourable barley traits into a more stable genetic background. Taking advantage of the breakage-fusion mechanism of univalent chromosomes, the ‘Rannaya’ winter wheat 7B monosomic line was used as female partner to the 7H addition line male, leading to the development of a compensating wheat/barley Robertsonian translocation line (7BS.7HL centric fusion, 2n = 42) exhibiting higher salt tolerance and elevated grain β-glucan content. Throughout the crossing programme, comprising the F_1_-F_4_ generations, genomic *in situ* hybridization, fluorescence *in situ* hybridization and chromosome-specific molecular markers were used to trace and identify the wheat and barley chromatin. Investigations on salt tolerance during germination and on the (1,3;1,4)-β-D-glucan (mixed-linkage glucan [MLG]) content of the seeds confirmed the salt tolerance and elevated grain MLG content of the translocation line, which can be directly applied in current wheat breeding programmes.

## Introduction

The exponential increase in the world population and the recent challenges imposed by the changing climate make it essential for crop breeding programmes to put the emphasis on developing higher-yielding cultivars with better nutritional parameters. Bread wheat (*Triticum aestivum*, 2n = 6x = 42), one of the most important crop species, is significantly affected by environmental stresses that seriously reduce its productivity [1,2]. Among the enviromental factors, soil salinization, which has been worsened by climate change, is one of the most constraining abiotic stresses threatening wheat yields. The breeding of salinity-tolerant, high- yielding varieties is therefore a valuable solution to ensure food security. This can be achieved *via* prebreeding programmes that transfer genes responsible for salt tolerance from related species into wheat. Barley (*Hordeum vulgare*, 2n = 2x = 14) is one of the most salt- tolerant crops, with significant variation among genotypes [3–5]. It can be cultivated on saline soil as it maintains growth despite accumulating Na^+^ in the leaves. Salt stress causes a complete metabolic rearrangement in the cells, affecting the sugar, amino acid and polyamine metabolism, and ion and redox homeostasis [6–8]. Similarly to other plant species, salt tolerance in barley is inherited as a polygenic trait with numerous quantitative trait loci (QTLs) mapped on several chromosomes including 7H [9,10].

Barley has a place in healthy diet, as the grain has a high content of (1,3;1,4)-β-D-glucan (MLG), a polysaccharide that helps to lower blood cholesterol and regulates blood sugar levels in humans [11,12]. The *cellulose synthase-like F6* (*HvCslF6*) gene that encodes the putative MLG synthase has been mapped to barley chromosome 7H [13, 14]. Located within the centromeric region of the long arm (7HL), the *HvCslF*6 gene has been proved to play a key role in controlling β-glucan biosynthesis, and its down-regulation results in decreased MLG within the endosperm [15,16].

Barley belongs to the tertiary genepool of wheat, so abiotic stress resistance and advantageous composition parameters can be transferred into wheat *via* wide hybridization. Crossing wheat with barley is challenging to achieve and the F_1_ hybrids can only be raised in embryo culture, due to the lack of the endosperm [17].

The Ukrainian six-rowed winter barley ‘Manas’ may carry new allelic variation compared to the two-rowed spring cultivar ‘Betzes’ (generally used in wheat-barley crosses) as it has better agronomic traits (e.g. abiotic stress tolerance and yield ability), and it is well adapted to Central European conditions [18]. A hybrid previously developed between the winter wheat ‘Asakaze’ and the barley cultivar ‘Manas’ was subsequently screened to select a series of disomic and ditelosomic addition lines carrying the individual chromosomes/chromosome arms from barley in the wheat background [18,19]. Addition lines make it possible to study the effect of a specific barley chromosome added to wheat. However, as aneuploids are genetically unstable, this requires continuous cytogenetic checks. Wheat/barley translocation lines carrying 42 chromosomes are mostly stable and are directly applicable in prebreeding programmes [20,21]. Furthermore, compensating translocations are particularly valuable, as the alien homoeologous chromosomes are better able to compensate for the loss of a wheat chromosome segment [22]. In a previous study the ‘Asakaze’/‘Manas’ 7H disomic addition (2n = 44) and 7HL ditelosomic addition lines (2n = 42+2 telocentrics) were characterized in detail for abiotic stress resistance and nutritional parameters. Germination tests performed under salt conditions and salt tolerance experiments in early development stages revealed higher salinity tolerance for both lines [19,23]. The addition of the barley chromosome 7H, carrying the gene responsible for MLG synthesis (*HvCslF6* gene), to the hexaploid chromosome set of wheat has been found to increase the MLG content of the grain [24].

The aim of the present study was to develop a stable, fully fertile compensating winter wheat/winter barley translocation line (2n = 42) directly amenable to wheat breeding programmes, which ultimately enhances salt tolerance and the MLG content of the grain. The breakage-fusion mechanism of univalent chromosomes was exploited to produce a new 7BS.7HL Robertsonian translocation line (RobT) and the new line was characterized by means of molecular genetic and cytogenetic methods (genomic *in situ* hybridization: GISH and fluorescence *in situ* hybridization: FISH), which confirmed the high salinity tolerance during germination and the higher MLG content of the grain, proving that 7BS.7HL RobT is a valuable genetic material readily applicable in wheat improvement.

## Material and methods

### Plant material

A crossing progamme was initiated in order to develop a compensating Robertsonian translocation in a wheat background, including the long arm of barley chromosome 7H, in order to obtain a stable, fully fertile genetic material carrying salt tolerance and elevated MLG content. A ‘Rannaya’ 7B winter wheat monosomic stock was pollinated with the previously generated ‘Asakaze’/‘Manas’ 7H disomic addition line. Double monosomics for wheat 7B and barley 7H chromosomes (carrying 42 chromosomes) were selected in the F_1_ generation using 7H-specific molecular microsatellite and STS markers and Feulgen staining. F_1_ plants were self-pollinated and descendants in the F_2_ generation were screened with specific markers for the 7HS and 7HL arms to detect individuals carrying the long arm of 7H. Plants with a monosomic Robertsonian translocation were identified in the F_3_ generation using GISH, and disomic (homozygous) wheat-barley centric fusions were selected in the F_4_ generation (S1 Fig). Fertility (number of seeds/spike) was assigned based on the main spikes of plants grown in phytotron growth cabinets and in the Martonvásár nursery in 2014–2015 and 2015–2016, respectively.

In phytotron experiments vernalisation was carried out at 4°C for 6 weeks, after which the vernalised plants were grown in 2 L pots filled with a 2:1:1 mixture of garden soil, humus and sand. The plants were grown until tillering under an initial regime of 15°C day: 10°C night temperature, 12 h light: 12 h dark photoperiod. The temperature was raised by increments of 2°C after tillering (day length 14 h), stem elongation (16 h illumination), flowering and 2 weeks after fertilization.

### Genomic *in situ* hybridization (GISH) and fluorescence *in situ* hybridization (FISH)

Mitotic chromosome spreads were obtained from germinating root tips as described by Lukaszewski [25]. GISH technique has been used to distinguish alien chromosomes from wheat chromosomes and wheat-alien translocated chromosomes in a wheat background [26, 27]. The GISH experiment was carried out using total genomic barley DNA labelled with a dig-nick-translation mix (Roche Diagnostics, Mannheim, Germany). Unlabelled wheat genomic DNA was used as a blocking agent at a ratio of 35:1. Probe detection was carried out with anti-digoxigenin-Rhodamine (Roche). Slides were mounted in Vectashield antifade solution (Vector Laboratories, Burlingame, USA) containing 2 μg·mL^-1^ 4ʹ-6-diamidino-2-phenylindole (DAPI). FISH was performed after rinsing the GISH hybridization signals off in 4 × saline-sodium citrate (4X SSC) Tween 20 (Sigma Aldrich, Darmstadt, Germany) at room temperature. Wheat-specific DNA repetitive probes (pSc 119.2, Afa family and pTa 71) [28–30] were used to identify the wheat chromosome arm involved in the Robertsonian translocation and to confirm the presence of the entire wheat genome (except the 7BL chromosome arm). The Afa family and pSc119.2 repetitive probes were amplified and labelled with PCR using digoxigenin-11-dUTP and biotin-16-dUTP, respectively, while the 45S rDNA clone pTa71 was labelled with 50% biotin-16-dUTP and 50% digoxigenin-11-dUTP by nick translation. Digoxigenin and biotin signals were detected with anti-digoxigenin-Rhodamine Fab fragments and streptavidin-FITC (Roche), respectively. The fluorescence signals were vizualized using a Zeiss Axioskop-2 fluorescence microscope (Zeiss, Oberkochen, Germany) equipped with filter sets appropriate for DAPI, FITC, Rhodamine and the simultaneous detection of FITC and Rhodamine (double filter set). Images were captured with a Spot CCD camera (Diagnostic Instruments, Sterling Heights, USA) and processed with Image Pro Plus software (Media Cybernetics, Silver Spring, USA).

### SSR and STS marker analysis

Genomic DNA was extracted from fresh young leaves of wheat cultivar ‘Asakaze’, barley cultivar ‘Manas’ and the wheat-barley RobT using Quick Gene-Mini80 (FujiFilm, Japan) with a Quick-Gene DNA tissue kit (FujiFilm, Japan) according to the manufacturer’s instructions. Barley chromosome arm-specific SSR markers (Bmac0031-7HS, HvID-7HL) [31] and the gene-specific HvCslF6 STS marker [32] were used to reveal the presence of the 7HS and 7HL barley chromosome arms. The 7BL chromosome arm-specific SSR marker (Wmc311-7BL) [33] was used to reveal the absence of the 7BL wheat chromosome arm. The primer pairs were tested on DNA templates containing genomic DNA from wheat cultivar ‘Rannaya’, the ‘Asakaze’/‘Manas’ 7H disomic addition line (7H), and the ‘Asakaze’/‘Manas’ RobT. PCR amplification was carried out under the conditions described by Cseh et al. [34]. PCR products were separated with a Fragment Analyzer Automated CE System equipped with a 96-Capillary Array Cartridge (Advanced Analytical Technologies, USA). The results were interpreted using PROsize v2.0 software (Advanced Analytical Technologies, USA).

### Morphological characterization of the 7BS.7HL RobT

The number of seeds per main spike was determined from 2 × 10 plants for each genotype (‘Rannaya’ wheat cultivar, 7H addition line and 7BS.7HL RobT) in experiments carried out in the Martonvásár phytotron and nursery.

### Evaluation of the salinity tolerance of the 7BS.7HL RobT

The salt stress response of the ‘Rannaya’ wheat genotype, the ‘Asakaze’/‘Manas’ 7H disomic addition line and 7BS.7HL RobT was evaluated after germination at the seedling stage. To determine the salt stress tolerance, 3×20 seeds from each genotype were surface-sterilized in 10% sodium hypochlorite for 15 minutes, rinsed twice in distilled water and germinated on wet filter paper in Petri dishes containing 0, 100, 150, 200 or 250 mM NaCl for 8 days (3 days in the dark and 5 days in the light) at a temperature of 25°C. The percentage of germinated seeds was determined after 5 days and the length of roots and coleoptiles was measured on the 8th day after germination.

### Evaluation of (1,3;1,4)-β-D-glucan content of the seeds of 7BS.7HL RobT

Four biological replicates of mature seeds (5 plants per replicate) were collected from the following genotypes: ‘Rannaya’ wheat cultivar, ‘Manas’ barley cultivar, 7H disomic addition line and 7BS.7HL RobT, and milled in a Retch M400 ball laboratory mill to produce wholemeal. Samples of finely ground grains of the plants (100 mg) were used for MLG analysis in four parallels according to AACC Method 32–23 [35] using a Megazyme kit (Megazyme, Bray, Ireland). The samples were suspended and hydrated in a buffer solution (pH 6.5) and then incubated with purified lichenase enzyme and filtered. An aliquot of the filtrate was then hydrolysed to completion with purified β-glucosidase. The quantity of produced D-glucose was determined using a glucose oxidase/peroxidase reagent.

### Statistical analysis

The fertility of 7BS.7HL RobT, the wheat cultivar ‘Rannaya’ and the 7H disomic addition line was compared pair-wise and differences in fertility were evaluated by means of Tukey’s post hoc test (SPSS 16.0) at the 0.05 significance level.

The data from the salinity tolerance experiment were also analysed using Tukey’s post hoc test (SPSS 16.0) at the 0.05 significance level.

Chemical data from the MLG experiment were evaluated using single-factor analysis in the Microsoft Excel program and were also analysed using Tukey’s post hoc test (SPSS 16.0) Values of P<0.05 were considered to be statistically significant.

## Results

### Development of the wheat-barley 7BS.7HL RobT

**Fig 1:**
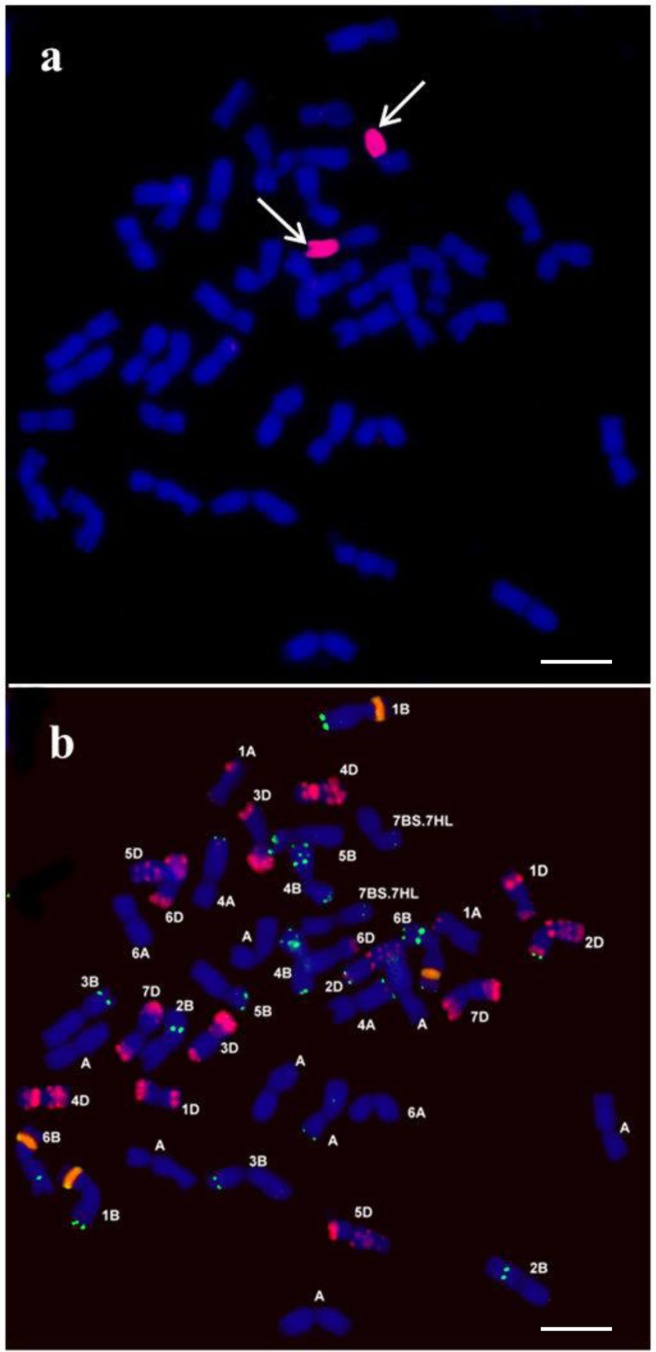
Genomic *in situ* hybridization and Fluorescence *in situ* hybridization on mitotic chromosomes of the 7BS.7HL Robertsonian translocation line. (a) Genomic *in situ* hybridization (GISH) on mitotic chromosomes of the 7BS.7HL Robertsonian translocation line (RobT). Labelled total genomic DNA of barley was used as probe and barley chromosome arm 7HL is highlighted in magenta (arrows). The chromosomes were counterstained with DAPI (blue). (b) Fluorescence *in situ* hybridization on mitotic chromosomes of 7BS.7HL RobT. DNA repetitive probes: Afa family (red), pSc119.2 (green), pTa71 (orange). Unidentified A genomic chromosomes are labelled by an ‘A’ letter. Scale bar = 10μm.

Earlier results revealed higher salt tolerance and MLG content in wheat-barley introgression lines carrying the 7H chromosome or 7HL arm [23,24]. In the present study the ‘Rannaya’ monosomic wheat line was crossed as female partner to the ‘Asakaze’/‘Manas’ 7H disomic addition line in order to induce rearrangements and develop a stable wheat/barley RobT carrying the favourable traits. Ten of the plants raised from 25 germinated F_1_ seeds were found to be monosomic for the 7B and 7H chromosomes when tested 7H with specific molecular markers and Feulgen staining. The F_1_ plants produced a total of 68 seeds with an average yield of below 10 seeds/plant, except for two individuals which yielded 21 and 22 seeds, respectively. From the 68 F_2_ seeds 66 germinated and these plants were screened for the presence of the 7HS or 7HL chromosome arms with the 7HS-specific Bmac0031 SSR marker and the 7HL-specific HvCSlF6 STS marker. The analysis identified two plants carrying only the 7HS barley chromosome arm and three plants containing solely the 7HL arm. Plants carrying either 7HS or 7HL were analysed in F_3_ with GISH in order to select progenies with wheat-barley Robertsonian translocations. Seventy-two F_3_ seeds were screened, of which 7 plants (9.72%) had the wheat-barley translocation (7BS.7HL). Ten other plants retained telocentrics either individually or in pairs, while three plants carried one barley isochromosome (7HS), suggesting the fusion of a telocentric pair. The barley chromatin was entirely eliminated from 52 of the F_3_ plants analysed. Individuals carrying the Robertsonian translocations were selfed and 50 F_4_ plants were screened by GISH (S1 Fig) of which three were found to be disomic lines (6%), 22 (44%) monosomic lines, 21 (42%) nullisomic lines and 4 (8%) plants carried a barley telocentric chromosomes. During the examination of disomic RobT lines the GISH results comfirmed the presence of 40 wheat chromosomes and the recombinant chromosome pair (7BS.7HL) (Fig 1A). Following random selection 49 F_5_ seeds originated from disomic plants were germinated and analysed by GISH and these were 100% disomic 7BS.7HL translocations.

**Fig 2:**
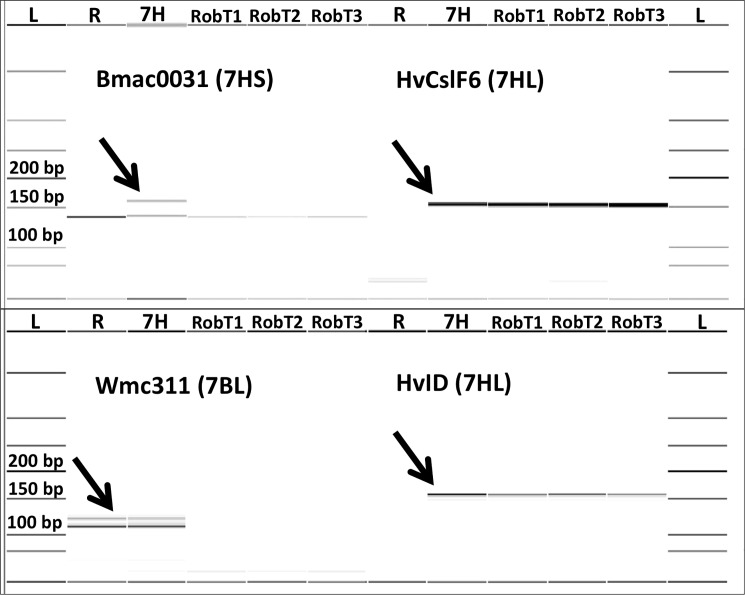
Capillary gel electrophoresis pattern of the 7H chromosome specific molecular markers and the 7BL chromosome arm-specific marker. Capillary gel electrophoresis pattern of the Bmac0031 (7HS), HvCslF6 (7HL) and HvID (7HL) 7H chromosome arm-specific molecular markers and the Wmc311 7BL chromosome arm-specific marker on the following DNA templates: ‘Rannaya’ wheat cultivar (R), ‘Asakaze’/‘Manas’ 7H disomic addition line (7H), and 7BS.7HL disomic Robertsonian translocation lines (RobT1, RobT2, RobT3). Chromosome arm-specific bands are indicated by arrows.

**Fig 3:**
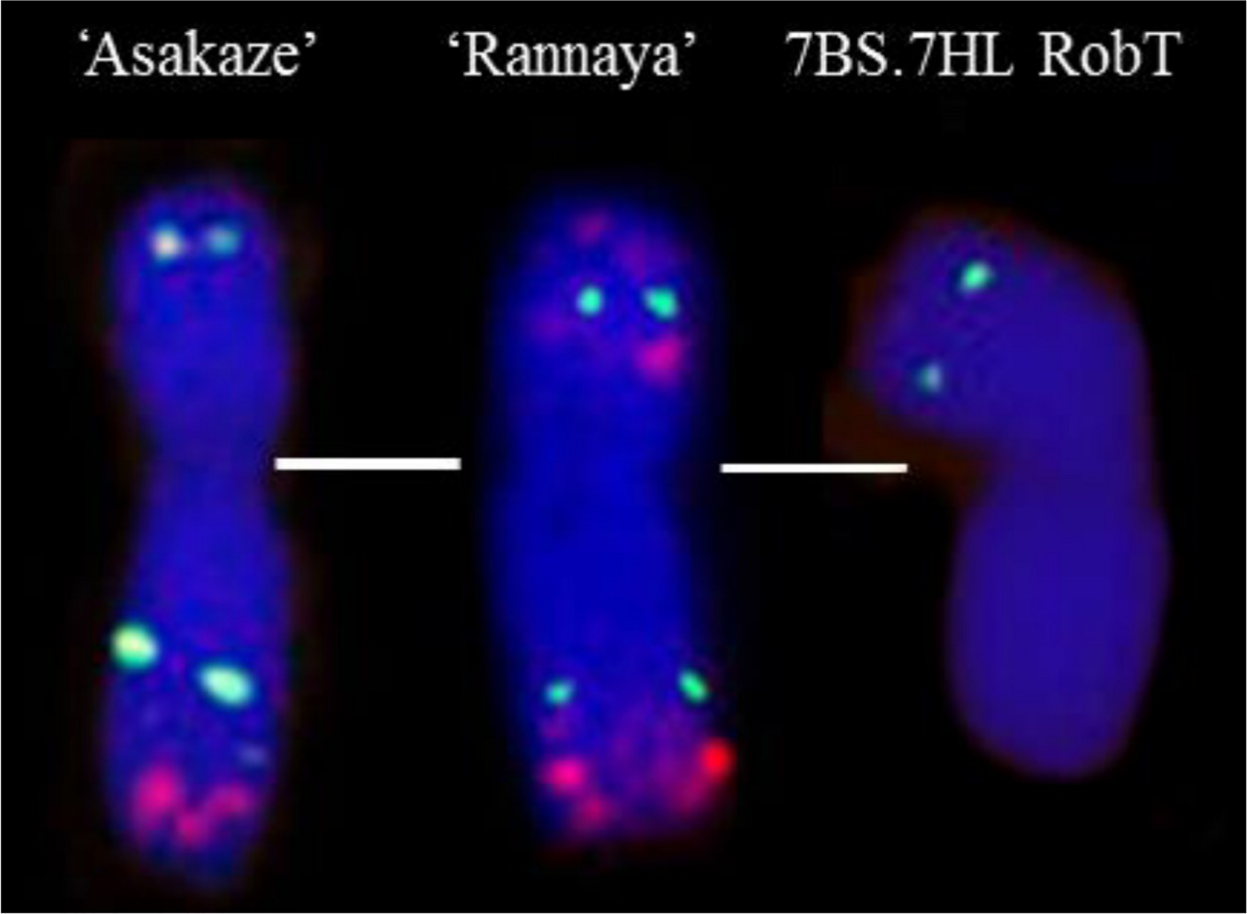
FISH pattern of chromosome 7B of the ‘Asakaze’ and ‘Rannaya’ wheat cultivars and the 7BS.7HL Robertsonian translocation chromosome. DNA repetitive probes: Afa family (red) and pSc119.2 (green).

The barley chromosome arms were identified by molecular marker analysis using the above specific molecular markers (Fig 2). The whole 7HL arm was detected by means of an STS marker located in the centromeric region of 7HL (HvCSLF6) and an SSR marker mapped to the telomeric region of the 7HL chromosome arm (HvID). After washing the GISH signals off, FISH was performed in order to verify the identity of the wheat chromosome arm taking part in the Robertsonian translocation. The translocated wheat chromosome arm exhibited only two subterminal pSc119.2 signals and was thus identified as 7BS (Fig 1B, Fig 3). The parental genotypes (‘Rannaya’ wheat, ‘Manas’ barley and the 7H disomic addition line used as control) and descendants of the disomic plants were maintained and multiplied in phytotron growth cabinets (F_5_) and in the Martonvásár nursery (F_6_-F_7_ generations).

### Morphological characterization of the 7BS.7HL RobT

**Fig 4:**
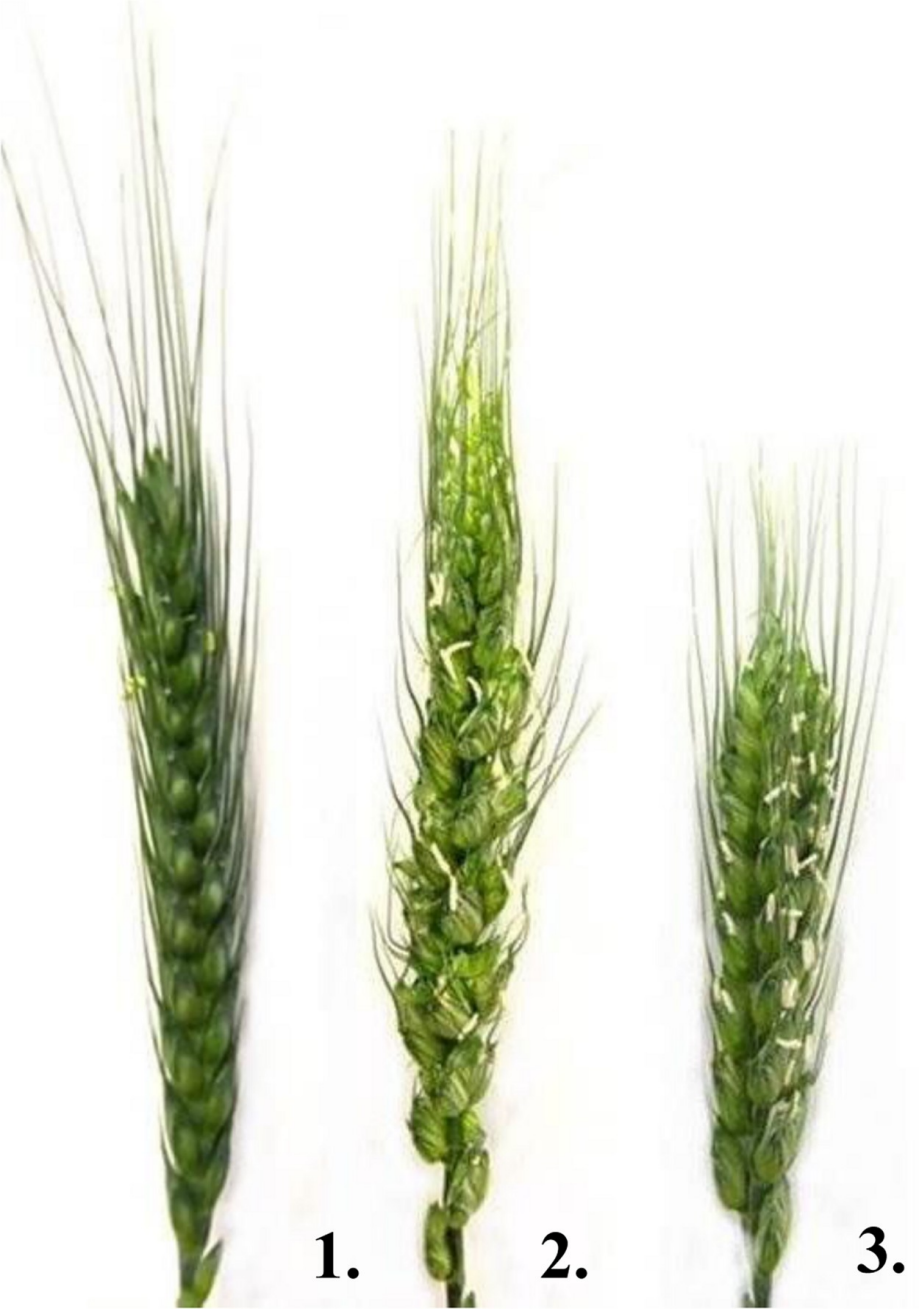
Phenotype of spikes of the parental genotypes and the 7BS.7HL centric fusion line. Phenotype of spikes of the parental wheat genotype ‘Rannaya’ (1), the 7H disomic addition (2) and the 7BS.7HL RobT line (3). Plants were grown in phytotron climate chambers.

The 7H addition line showed low fertility, especially in the apical part of the spike, under both phytotron and field conditions. On the other hand, the fertility of the RobT was similar to that of the ‘Rannaya’ winter wheat cultivar, demonstrating that the crossing strategy had sucessfully restored the fertility of the 7H addition (S2 Fig). The significant improvement in fertility was reflected by the fact that although the spikes of the RobT were shorter (Fig 4) the number of seeds/spike was twice as high for the RobT than for the 7H addition line.

### Salt stress response of 7BS.7HL RobT

The germination % and the root and shoot growth of the ‘Rannaya’ wheat cultivar, the 7H addition line and the 7BS.7HL RobT were determined after germination in NaCl solutions with various concentrations (0, 100, 150, 200 and 250mM). These results are presented in Table 1.

**Table 1:** Germination percentage of the genotypes analysed.

	0 Mm NaCl	100 Mm NaCl	150 Mm NaCl	200 Mm NaCl	250 Mm NaCl
**Wheat cv. ‘Rannaya’**	100^a^	86^bc^	73^de^	46^f^	30^g^
**7H addition line**	100^a^	100^a^	85^bc^	78^d^	70^e^
**7BS.7HL RobT**	100^a^	100^a^	90^b^	80^cd^	75^de^

Values are means ±SD of 3 x 20 replicates per genotype. Different letters indicate significant differences at P < 0.05 using Tukey’s post hoc test.

All three genotypes germinated well without salt treatment and the germination percentage decreased slightly (86%) in ‘Rannaya’ under the mild salt stress induced by 100mM NaCl. Above this NaCl concentration, the germination percentage decreased, especially in ‘Rannaya’. When treated with 150, 200 mM and 250 mM NaCl, the germination capacity of the wheat genotype dropped considerably (to 73%, 46% and 30%, respectively). In contrast, both the 7H addition line and the RobT maintained a very high germination rate when subjected to 150, 200 and 250 mM NaCl (85%, 78% and 70% for the 7H addition line and 90%, 80% and 75% for RobT, respectively).

**Fig 5:**
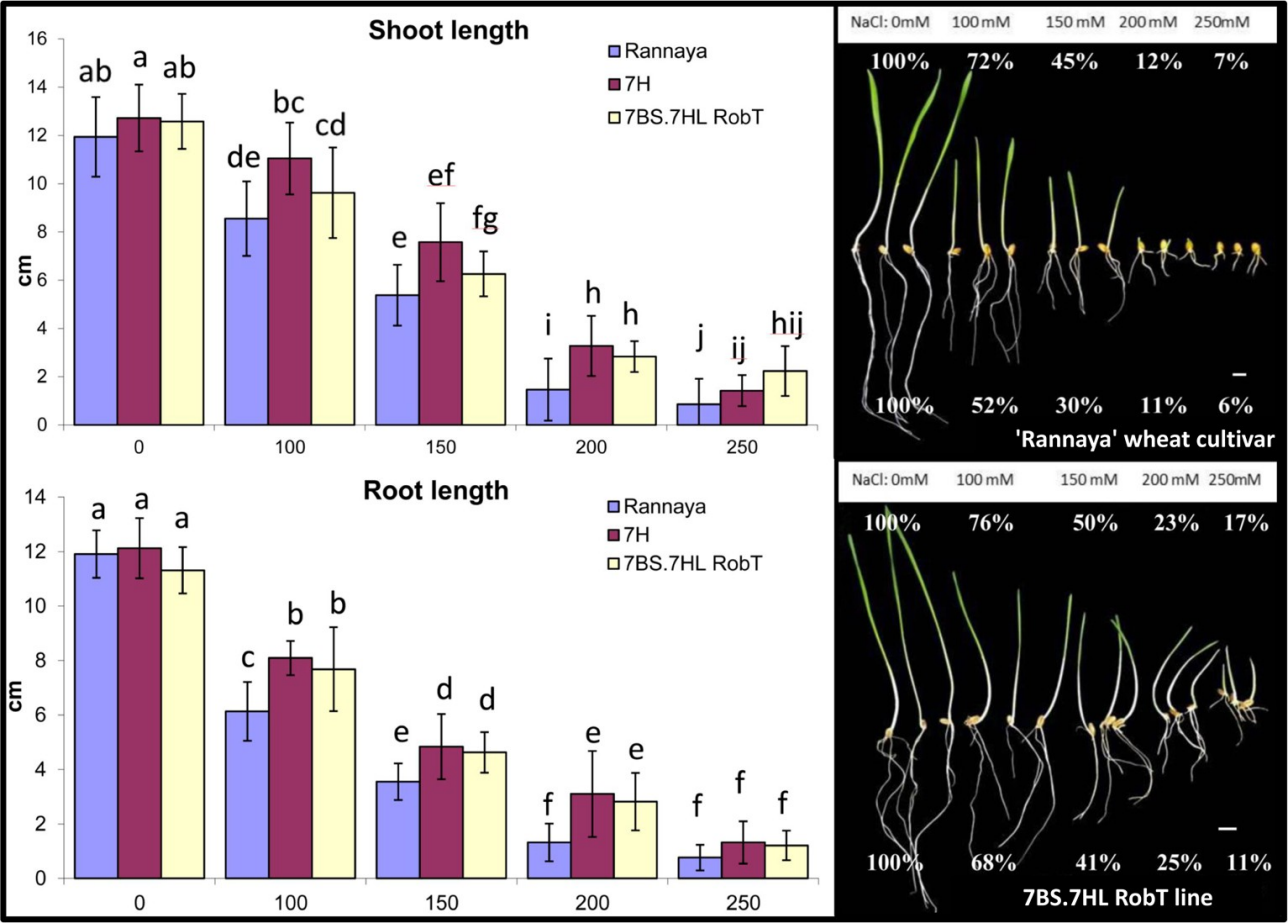
Root and shoot length measured at five levels of NaCl treatments. Root and shoot length measured on the 8th day after germination for wheat cultivar ‘Rannaya’, the ‘Asakaze’/‘Manas’ 7H disomic addition line and 7BS.7HL RobT at five levels of NaCl treatments (0, 100, 150, 200 and 250 mM solution). Different letters indicate significant differences at P < 0.05 using Tukey’s post hoc test. The pictures are represented the salt tolerance of the wheat parent (‘Rannaya’) and 7BS.7HL RobT during germination at different NaCl concentrations (0–250 mM). Percentages show the inhibition of root and shoot growth compared to the controll (0 mM NaCl). The reduction of the germination capacity and the inhibition of root and shoot growth were greater in wheat parent ‘Rannaya’ than in the 7BS.7HL line. Scale bar = 1cm.

The salt tolerance of the ‘Rannaya’ wheat cultivar, the ‘Asakaze’/‘Manas’ disomic addition line and 7BS.7HL RobT was also investigated by measuring the root and shoot lengths on the 8th day after germination. Germinating the seeds in 100, 150, 200 or 250 mM NaCl solution resulted in a decrease of root and shoot coleoptile length as a % of the control (Fig 5). The growth reduction was more pronounced in ‘Rannaya’ than in the addition and the RobT. For instance, the roots of the 7BS.7HL RobT were twice as long as those of the wheat parental line when treated with 200 mM NaCl (Fig 5). The differences between the genotypes were more pronounced for the roots than for the shoots. The differences in shoot lengths were not as significant due to the higher standard deviation (Fig 5). Salt stress reduced the growth vigour of all the genotypes during the first 4 days, compared to that of the control plants (without NaCl treatment), but the introgression lines showed better growth during the subsequent 4 days and exhibited better growth vigour, resulting in greater differences between the genotypes (Fig 5).

### Analysis of the MLG content of 7BS.7HL RobT

**Fig 6:**
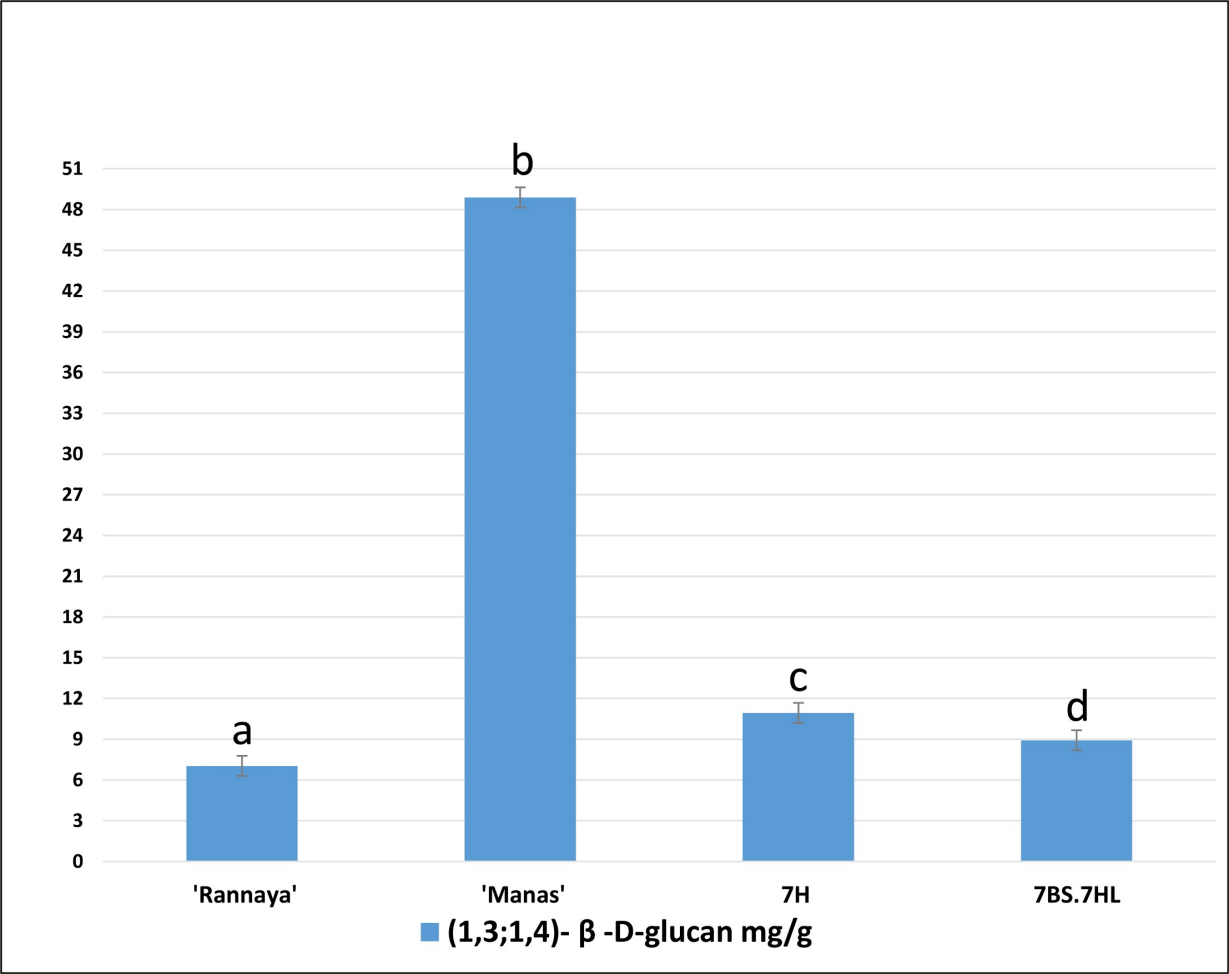
(1,3;1,4)-β-D-glucan content in the grains. Mixed-linkage β-glucan content in the grains of wheat (‘Rannaya’), barley (‘Manas’), the 7H disomic addition line (7H) and 7BS.7HL RobT (7BS.7HL). Mixed-linkage β-glucan analysis was performed in four parallels according to AACC Method 32–23. Different letters indicate significant differences at P < 0.05 using Tukey’s post hoc test. Bars show the LSD 5% value: 0.74.

The mean MLG levels in four biological replicates of the studied genotypes are presented in Fig 6, together with the LSD 5% values. The mean MLG level (mg/g dry matter) of the ‘Rannaya’ wheat cultivar was 7.04 mg (0.7%), while that of the barley cultivar ‘Manas’ was seven times as high (48.89 mg/g, 4.89%). The β-glucan content of the seeds was 8.93 mg/g (0.9%) for 7BS.7HL RobT and 10.93 mg/g (1.1%) for the 7H addition line, so the MLG level of the 7BS.7HL RobT, carrying the *HvCslF6* gene, exceeded the β-glucan content of wheat by 26.85%.

## Discussion

The ‘Asakaze’/’Manas’ winter wheat-winter barley 7BS.7HL compensating RobT was developed with the aim of transferring agronomically favourable genes from cultivated barley to bread wheat. The present work demonstrated that a stably inherited Robertsonian translocation comprising the 7HL barley chromosome arm in a wheat background could effectively enhance the salintity tolerance of the wheat plant and increase the MLG content of the grain.

Wheat-alien amphiploids and addition lines cannot be directly used in wheat breeding programmes because of their genetic instability. On the other hand, stably inherited translocation lines carry a subchromosomal segment of an alien chromosome integrated into the wheat genome and usually comprise 42 chromosomes. A number of wheat-ancestral Robertsonian translocations were reported involving wild relatives of wheat, such as *Dasypirum villosum* [36], *Haynaldia villosa* [37], *Thinopyrum bessarabicum* [38], *Thinopyrum intermedium* [39], *Aegilops searsii* [40] and *Aegilops speltoides* [41]. For instance, the stripe rust resistance gene *Sr59* was introgressed into hexaploid wheat from rye through a compensating 2DS.2RL Robertsonian translocation [42]. One of the most well-known and widely applied ancestral introgressions was the transfer of the 1RS rye chromosome arm from the Russian cultivars ‘Aurora’ and ‘Kavkaz’ into the wheat background as a compensating 1BL.1RS translocation [43, 44]. Consequently, 1BL.1RS is present in more than 1000 wheat cultivars worldwide and contributed to improving the yield potential, adaptability and biotic stress resistance of wheat [45,46].

Only a few wheat-barley compensating translocation lines are so far available for wheat improvement. Koba et al. [47] reported the development of a 5HS.5BL RobT in addition to the 42 wheat chromosomes from a cross between the ‘Schinchunaga’ wheat and ‘Nyugoruden’ barley cultivars. Danilova et al. [48] produced a complete set of homoeologous group 7 compensating translocations introducing chromosome arms from the ‘Betzes’ spring barley variety into the ‘Chinese Spring’ wheat background. The ‘Manas’ barley used in the present study is a six-rowed winter variety having good agronomic traits (high yield potential, winter hardiness, salt tolerance, high MLG content) [18]. Similarly, the ‘Rannaya’ wheat used as female parent in the cross is a cultivated winter wheat with high yielding potential, making it more suitable for breeding purposes.

Depending on the wheat and alien chromosomes involved and the environmental conditions, the desired compensating wheat-alien Robertsonian translocations can be recovered at variable frequencies, ranging from low (1.77%) to fairly high (20%) [36,48–50]. In this study the efficiency of the breakage-fusion mechanism was 7.6% in the F_2_ generation. Besides the crossing procedure and the detailed genetic investigation, this study focused on the phenotyping of the newly developed plant material. Wheat is not tolerant to high level of soil salt content. King et al.[51] reported the possibility of transferring salt tolerance genes from a wild relative (*Thinopyrum bessarabicum*) into bread wheat. In a previous study, germination tests performed under salt conditions and salt tolerance tests during early development stages confirmed the higher salinity tolerance of lines carrying the 7H barley chromosome or the 7HL arm compared to wheat and other wheat-barley addition lines (2H, 3H, 4H, 6H and 7HS) [19,23]. Detailed physiological, biochemical and molecular genetic investigations related to typical salt tolerance mechanisms revealed that Na^+^ uptake and transport are not responsible for the elevated salt tolerance of the 7H and 7HL addition lines. The higher salt tolerance was mainly due to the improved osmotic adjustment capacity of these lines [52]. Osmotic adjustment is one of the mechanisms contributing to the tolerance of salt-stressed plants [53]. QTLs associated with genes responsible for the relative water content (RWC) and water-soluble carbohydrate concentration (WSC) of plants were identified and mapped using composite interval mapping to barley chromosome arm 7HL [54]. The *bSS1B* gene is associated with a QTL for RWC encodes barley sucrose synthase, which is a key enzyme in the carbohydrate metabolism, catalysing fructose and UDP-glucose synthesis [55,56], while the *Acl3* gene encodes a co-factor protein associated with a QTL for RWC on the 7HL arm, having a role in membrane protection during stress [54].

Earlier experiments revealed that the improved salt tolerance of the 7H addition line was due to osmotic adjustment achieved through proline accumulation and enhanced soluble carbohydrate metabolism [52]. The similar experimental design used in the present experiments showed that both the ‘Asakaze’/‘Manas’ 7H disomic addition line and the 7BS.7HL RobT performed better under salt stress conditions than the wheat parental cultivar. Barley cv. ‘Manas’ and the 7HL ditelosomic addition thus gave results similar to those observed earlier for the 7H addition line, suggesting that the enhanced salt tolerance observed for the 7BS.7HL translocation line also operates *via* osmotic adjustment [52]. The slow root and shoot growth during the first 4 days of salt treatment and the subsequent acceleration further support this assumption, as the reprogramming of the carbohydrate metabolism and the accumulation of large pools of soluble sugars require several days. However, further investigations are needed to confirm the role of sugars in the salt tolerance mechanisms operating in the 7BS.7HL RobT line.

The seed MLG content of the ‘Manas’ barley cultivar is seven times as high as that of ‘Rannaya’ wheat. The presence of the 7HL barley chromosome arm increased the MLG content of the seeds by 26.83%, demonstrating that the *HvCslF6* gene responsible for its synthesis is functional in the wheat genetic background. Recent QTL and genome-wide association studies suggested that other proteins may also play a role in (1,3;1,4)-β-D-glucan biosynthesis, implying that the introduction of other genes from barley would result in higher dietary fibre content in wheat. Canditate genes were mapped to the 1HS, 2HL, 3HS, 3HL, 4HL, 5HS, 6HS and 7HS chromosome arms [57–59]. The current model for (1,3;1,4)-β-D-glucan biosynthesis proposes that the *Hv*CSLF6 enzyme is only responsible for creating the β-(1,4)-glucosidic linkages, which are then joined together to produce the mixed-linkage glucan through a β-(1,3)-glucosidic linkage catalysed by an as yet unidentified protein [60]. The higher MLG content of the 7H addition line compared to the RobT thus suggests that the QTLs mapped on 7HS and 7HL may have an additive effect and that the presence of both groups of loci results in a higher MLG level (Fig 6).

Besides the traits characterized in this study the 7HL chromosome arm carries other genes of breeding interest. For instance, a locus (Rps6) conferring stripe rust (*Puccinia striiformis*) resistance was likewise mapped to the distal region of 7HL in the barley cultivar ‘Tamalpais’ and the barley line ‘Y12’ [61]. The wheat-barley RobT produced in the present work thus has the potential to improve stripe rust resistance in wheat.

The ‘Ranaya’/‘Manas’ 7BS.7HL compensating RobT carries 42 chromosomes, and genomic *in situ* hybridization revealed a high degree of chromosome stability, implying that the introgressed agronomic characters are stably inherited. Moreover, the fertility of the 7BS.7HL RobT was similar to that of wheat, indicating that the infertility of the parental 7H addition line had been successfully overcomed. The good yield potential and fertility of the RobT makes it possible to use this line directly in wheat breeding programmes to improve biotic and abiotic stress resistance and to confer better nutritional parameters on cultivated bread wheat.

## Conclusions

The introgression of barley chromatin segments into wheat, as in the stably inherited whole arm Robertsonian translocation developed in the present study, widens the genetic diversity of cultivated bread wheat. Evidence was provided that functional genes originating from the long arm of barley chromosome 7H improved the salinity tolerance and increased the (1,3;1,4)-β-D-glucan content of the seed in the newly developed ‘Asakaze’/‘Manas’ 7BS.7HL introgression line. The genome stability and fertility of the compensating translocation line make it a suitable genetic material for direct application in wheat breeding programmes.

## Supporting information

S1 Fig Crossing strategy applied for the development of 7BS.7HL RobT. (DOCX)

S2 Fig Number of kernels/main spike of the 7BS.7HL RobT, ‘Rannaya’ wheat cultivar and ‘Asakaze’/‘Manas’ 7H disomic addition line. Number of kernels/main spike of ten randomly selected plants (1–10) in Martonvásár phytotron and field experiments for the 7BS.7HL RobT and for the ‘Rannaya’ wheat cultivar and ‘Asakaze’/‘Manas’ 7H disomic addition line used as control. Different letters indicate significant differences at P < 0.05 using Tukey’s post hoc test.
(DOCX)
