# Supplementary material for: Development of a new 7BS.7HL winter wheat-winter barley Robertsonian translocation line conferring increased salt tolerance and (1,3;1,4)-β-D-glucan content
Source: PLoS One. 2018 Nov 5;13(11):e0206248. doi: 10.1371/journal.pone.0206248 (PMC6218033; doi:10.1371/journal.pone.0206248)
Supplement: S1 Fig — (DOCX) [file pone.0206248.s001.docx]

*Triticum aestivum* cv. ʻRannajaʼ × ʻAsakazeʼ/ʻManasʼ

7B monosomic stock wheat/barley 7H

disomic addition line

(20ʺ+7Bʹ+7Hʹ) F_1_  Double monosomic plants

were selected using Feulgen

staining and molecular markers

Self pollination

F_2_ Plants that had only the

7HS or 7HL arm were selected

Self pollination using molecular markers

F_3_ 7BS.7HL translocation lines

were identified using molecular

markers and GISH

Self pollination

Plants homozygous for the F_4_

7BS.7HL translocation were

selected using GISH and FISH

S1 Fig. Crossing strategy applied for the development of 7BS.7HL RobT
