# Supplementary material for: Development of a new 7BS.7HL winter wheat-winter barley Robertsonian translocation line conferring increased salt tolerance and (1,3;1,4)-β-D-glucan content
Source: PLoS One. 2018 Nov 5;13(11):e0206248. doi: 10.1371/journal.pone.0206248 (PMC6218033; doi:10.1371/journal.pone.0206248)
Supplement: S2 Fig — Number of kernels/main spike of ten randomly selected plants (1–10) in Martonvásár phytotron and field experiments for the 7BS.7HL RobT and for the ‘Rannaya’ wheat cultivar and ‘Asakaze’/‘Manas’ 7H disomic addition line used as control. Different letters indicate significant differences at P < 0.05 using Tukey’s post hoc test. (DOCX) [file pone.0206248.s002.docx]

S2 Fig. Number of kernels/main spike of ten randomly selected plants (1-10) in Martonvásár phytotron and field experiments for the 7BS.7HL RobT and for the ʻRannayaʼ wheat cultivar and ʻAsakazeʼ/ʻManasʼ 7H disomic addition line used as control. Different letters indicate significant differences at P < 0.05 using Tukey’s post hoc test.
